# Ion-retention properties of graphene oxide/zinc oxide nanocomposite membranes at various pH and temperature conditions

**DOI:** 10.1038/s41598-024-51309-y

**Published:** 2024-01-16

**Authors:** Amir Hassanpour, Marc A. Gauthier, Shuhui Sun

**Affiliations:** Institut National de la Recherche Scientifique, Centre Énergie Matériaux Télécommunications, Varennes, QC J3X 1P7 Canada

**Keywords:** Mechanical and structural properties and devices, Nanoparticles

## Abstract

Laminar graphene oxide (GO) is a promising candidate material for next-generation highly water-permeable membranes. Despite extensive research, there is little information known concerning GO's ion-sieving properties at high acidic/basic pH and temperatures. In this study, the ion-blockage properties of the pristine GO and GO/zinc oxide (ZnO) nanocomposite membranes were tested using a non-pressure-driven filtration setup over a wide range of pH and temperatures. The ZnO nanoparticles within the composite membranes were synthesized via the room-temperature oxidation of zinc acetate and zinc acrylate precursors and were uniformly distributed across the composite membrane. It is observed that partially replacing the zinc acetate precursor with zinc acrylate improves the blockage performance of the composite membranes under extreme basic conditions by 42%. Moreover, photocatalytically-reduced composite membranes blocked copper sulfate ions 28% more than as-prepared composite membranes. Further, it was discovered that the composition of the membrane plays a vital role in its ion blockage performance at higher temperatures.

## Introduction

The fast permeation of water molecules through multilayer graphene oxide (GO) membranes makes them excellent candidates for next-generation water filter materials^1–4^. The rate of water permeation through a laminar GO membrane is 100 times higher than that of commercial ultrafiltration membranes under external pressure^5^. It has been shown that solutes with diameters larger than ~ 4.7 Å are blocked by these membranes, while water molecules are able to pass through nanoscale channels within the membrane^6^. On the other hand, pristine GO has had limited success for desalination since the hydrated diameters of most monovalent and divalent ions are smaller than the cut-off limit of the membrane^6,7^. The poor stability of GO membranes, particularly in wet conditions, is another pitfall of this technology^8–10^. Extensive efforts have been undertaken to tackle these drawbacks by adding organic and inorganic additives to pristine GO membranes^5,9,11–13^. Despite some success^14,15^, the described composite membranes' ionic rejection performance in specific application conditions such as low/high pH and elevated temperature remains unclear.

Among all inorganic additives, zinc oxide (ZnO) nanoparticles stand out due to their positive impact on filtration and beneficial antimicrobial properties^16–18^. More specifically, GO/ZnO composite membranes have five times higher water permeability than pristine GO membranes, while maintaining the same rejection performance for dye molecules (1–2 nm)^19^. To the extent of our knowledge, ZnO nanoparticles are the only inorganic nanoparticles that can be synthesized in a non-acidic medium at room temperature without affecting the level of oxidation of the GO flakes within the membrane. In addition, the integration of ZnO nanoparticles into the GO membrane imparts microbicidal properties, which reduce the potential risk of biofouling^17,20^. To fine-tune and explore how the interactions between the ZnO nanoparticles and the GO flakes within the membrane influence the physical properties of the composite membranes, two different ZnO precursors were employed for their in situ synthesis: zinc acrylate and zinc acetate. These acrylate/acetate ligands are expected to influence the hydrophilicity, affinity for ZnO nanoparticles for GO, pH sensitivity, and mechanical robustness of the resulting membranes. The ability of these membranes to block copper sulfate as a model solute is studied under a wide range of pH conditions and temperatures.

To understand the rudimentary ion rejection properties of GO and GO/ZnO composite membranes under different filtration conditions, this study employs a custom diffusion cell similar to that reported previously^6,7,21,22^. The setup has two double jacket chambers that are either filled with a copper sulfate solution or distilled water (feed and permeate, respectively). Copper sulfate was chosen because, in a previous experiment^7^, the pristine GO membrane was nearly impermeable to these ions for at least three hours. The two solutions touch opposite sides of the membrane and the driving force for filtration is osmotic pressure, due to imbalanced ion concentrations and nano-capillary forces within nanochannels that exist in the membrane. The membrane blockage capacity is obtained by comparing the conductivity of the feed and permeate after the intended period of exposure. Due to the absence of an external force in this setup, the unpredictable damage that usually adversely affects the rejection performance of GO membranes is expected to be minimized. Furthermore, the double jacket chamber enables the measurement of the ion-retention performance of the membranes at elevated temperatures. A comprehensive overview of the GO/ZnO composite membrane's ion-retention performance is presented, over a wide range of pH, temperature, and over an extended time period that goes beyond existing data in the literature. Moreover, control of ion permeability by photoreduction of the GO/ZnO membranes using UV irradiation is reported for the first time.

## Results

**Figure 1:**
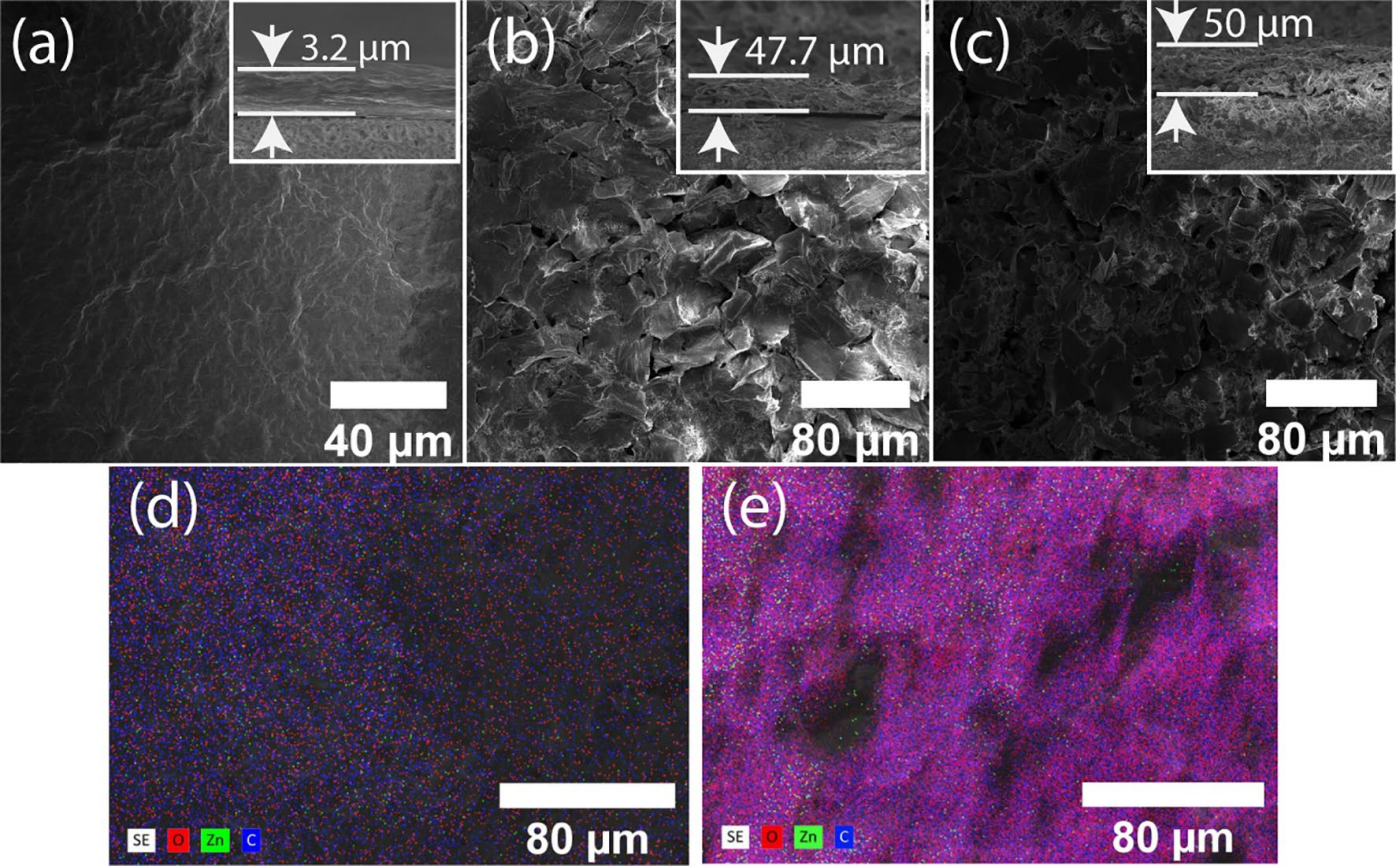
Representative SEM images of (**a**) pristine GO, (**b**) Composite 0, and (**c**) Composite 20 membranes. The insets show the cross-section and thickness of each membrane. EDS images of the (**d**) Composite 0 and (**e**) Composite 20 membranes show a uniform distribution of the ZnO nanoparticles (green dots) over the membrane.

The surface and cross-sectional SEM images of the GO and Composite 0–20 membranes are shown in Fig. 1a–c. The pristine GO membrane has a smooth contiguous surface, while the composite membranes consist of bumpy disintegrated flakes with some randomly distributed voids on the surface and throughout the bulk. The thickness of the GO, Composite 0, and Composite 20 membranes are 3.2 µm, 47.7 µm, and 50 µm, respectively. Hence, the intercalation of the ZnO nanoparticles significantly enlarged the thickness of the membranes relative to pristine GO, whereas replacing zinc acetate with zinc acrylate had a minimal effect on thickness. Elemental analysis of the Composite 0–20 membranes shows a uniform distribution of the ZnO nanoparticles (green dots; Fig. 1d,e). Increasing the percentage of the zinc acrylate precursor had no observable effect on the appearance and thickness of the membranes.

**Figure 2:**
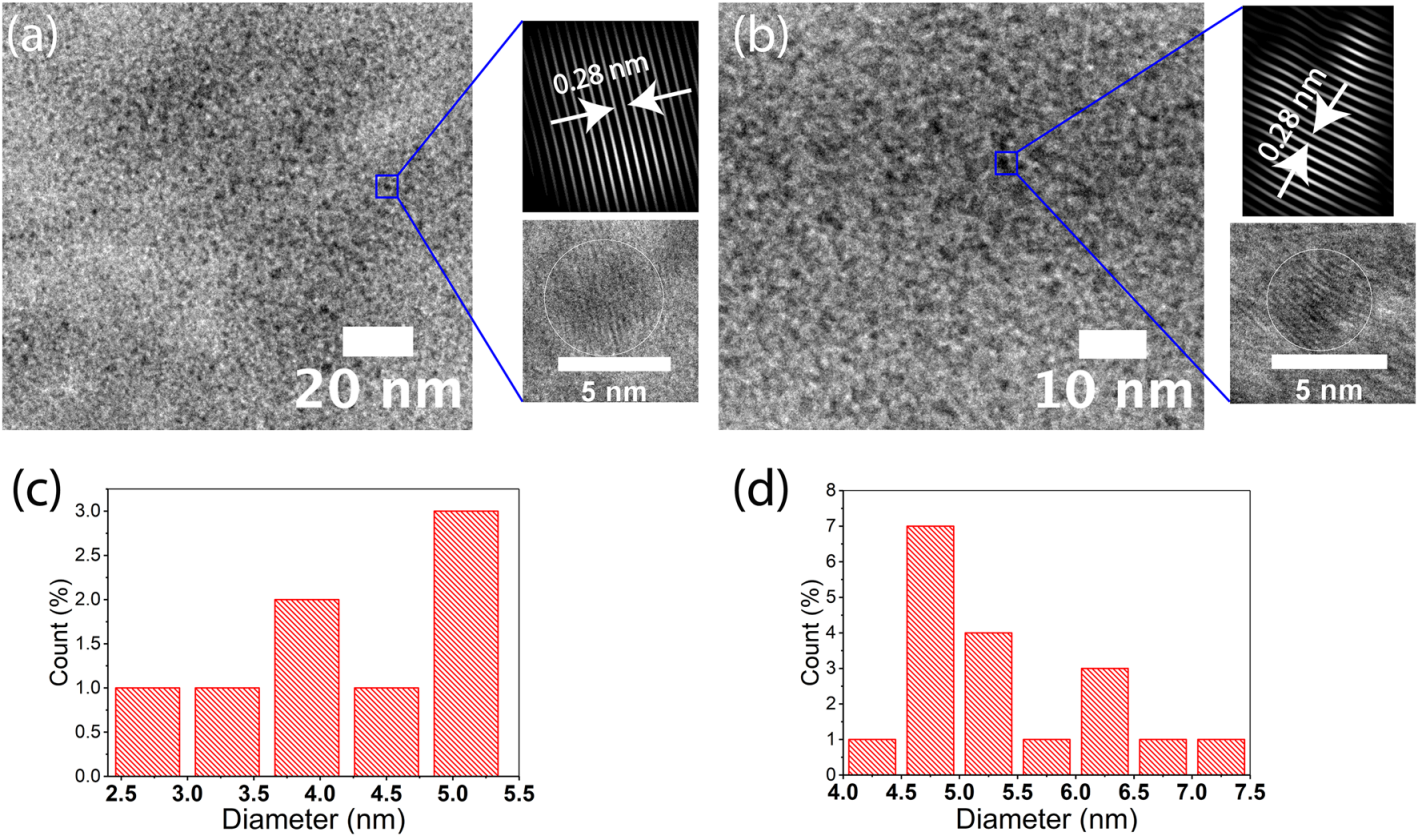
The low-resolution TEM images of the (**a**) Composite 0 and (**b**) Composite 20 membranes show the uniform distribution of the ZnO nanoparticles (black dots) over the blanket of GO flakes. The insets show the high-resolution TEM images of a spherical ZnO nanoparticle and its atomic spacing. The ZnO nanoparticle size distribution of the (**c**) Composite 0 and (**d**) Composite 20 membranes.

To analyze the size distribution of the ZnO nanoparticles embedded within the Composite 0–20 membranes, TEM was used. Figure 2a,b show that ZnO nanoparticles (black dots) are uniformly distributed within both membranes. The insets show the fast Fourier transform patterns of spherical ZnO nanoparticles with a d-spacing of 0.28 nm. The size distribution of the ZnO nanoparticles in the Composite 0 and Composite 20 membranes is shown in Fig. 2c,d, respectively. The average diameter of the ZnO nanoparticles in the Composite 0 membrane is slightly smaller than the counterparts in the Composite 20 membranes, and no obvious normal distribution can be seen due to random nucleation. The diameter of ZnO nanoparticles ranges from 2 to 5 nm for the Composite 0 and from 4 to 7 nm for the Composite 20 membranes, respectively.

**Figure 3:**
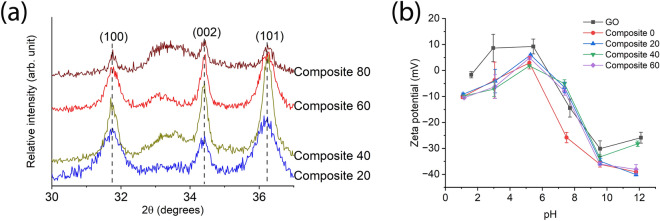
(**a**) XRD patterns of the composite 20–80 membranes. The identified peaks correspond to lattice planes of the ZnO wurtzite crystal. (**b**) Zeta potential of the as-prepared aqueous dispersions of the pristine GO and Composite 0–60 membranes versus pH. Data presented as mean ± SD, n = 3. In both panes, the percentages refer to the amount of zinc acrylate in the feed used to prepare the ZnO nanoparticles.

The successful synthesis of the Composite 0 membrane using zinc acetate as a precursor has been previously reported^19^. The formation and intercalation of the ZnO nanoparticles within the Composite 20–80 membranes were verified by XRD and the patterns are shown in Fig. 3a. The peaks at 31.7°, 34.4°, and 36.2° are the three main peaks of the ZnO wurtzite crystal structure. The XRD patterns indicate that the zinc acetate can be partially substituted with zinc acrylate without affecting the crystallization of the ZnO nanoparticles, which are prepared by the room temperature oxidation method. Zinc acrylate could be incorporated to a maximum of 80%, however, the resulting Composite 80 membranes were very brittle and could not withstand the permeation tests. Therefore, this membrane, as well as higher degrees of substitution with zinc acrylate, were not further characterized in this work.

The zeta potential of the aqueous dispersions of pristine GO and Composite 0–60 material versus pH is shown in Fig. 3b. The zeta potential of the pristine GO dispersion is slightly positive at a slightly acidic pH, though becomes negative above pH 6. The Composite 0–60 dispersions followed the same trend, albeit the zeta potentials tended to be slightly more negative than those of pristine GO at the more extreme acidic and basic pH conditions.

**Figure 4:**
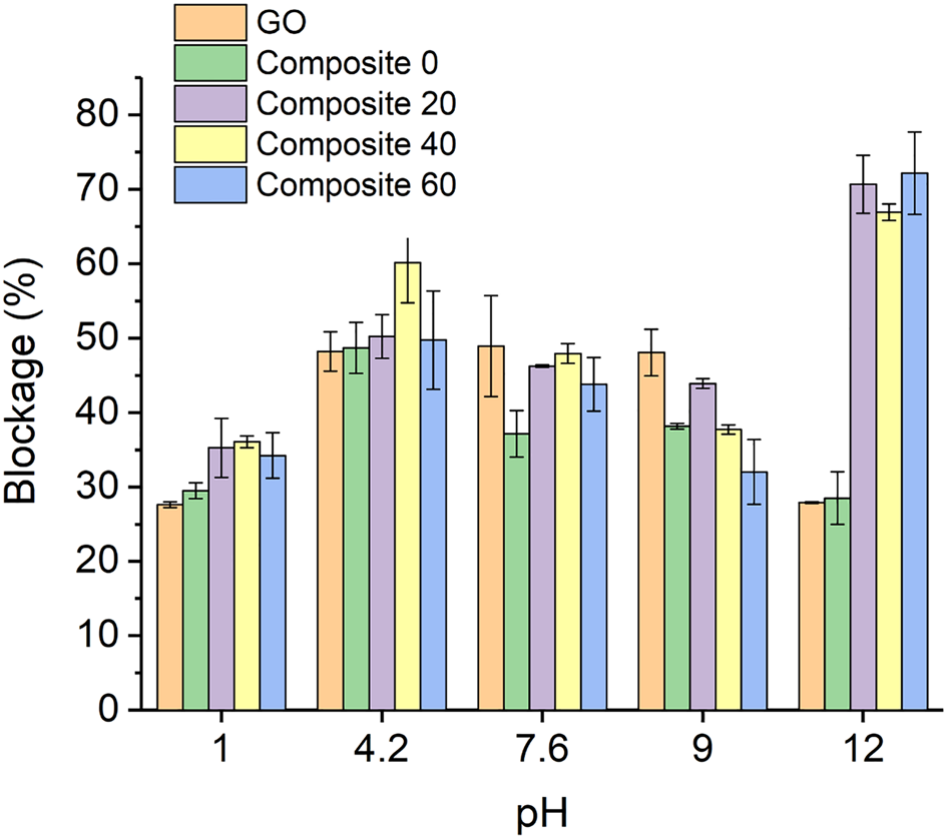
Ion blockage percentage of the pristine GO and Composite 0–60 membranes against copper sulfate feed solution measured at room temperature for 24 h at different pH values as mean ± SD, n = 3.

The ion blockage percentage of the GO and composite membranes are depicted in Fig. 4, which provides a good overview of the salt filtration ability of these membranes under different pH conditions. The membrane blockage percentage consists of the ability of the membrane to reject and adsorb the solutes. Pristine GO membranes block ~ 49% of ions in the pH range 4.2–9, while the blockage is reduced by approximately half this value in extremely acidic and basic conditions (pH 1 and pH 12). The presence of ZnO nanoparticles within the GO membrane generally did not greatly change this trend, except at highly alkaline pH. Curiously, the Composite 20–60 membranes behaved very differently from the others at pH 12. As shown in Fig. 4, at pH 12, ion blockage increased from ~ 30% for pristine GO and Composite 0 membranes to 70% for Composite 20–60 membranes. A greater blockage was observed when the zeta potential was near neutral, while it decreased at pH values where the zeta potential deviated from neutrality. This result suggests that the membranes differ in terms of hydrophilicity and potentially their swelling, influencing inter-sheet spacing, in a pH-dependent manner. Nevertheless, the magnitude of change to blockage performance did not scale with the magnitude of changes observed to zeta potential of the aqueous dispersion of the membrane material. The total volume of the feed and permeate water decreased after each test due to absorption by the membrane. However, there was no direct correlation between the volume of solution absorbed by the membranes and their corresponding blockage percentage.

**Figure 5:**
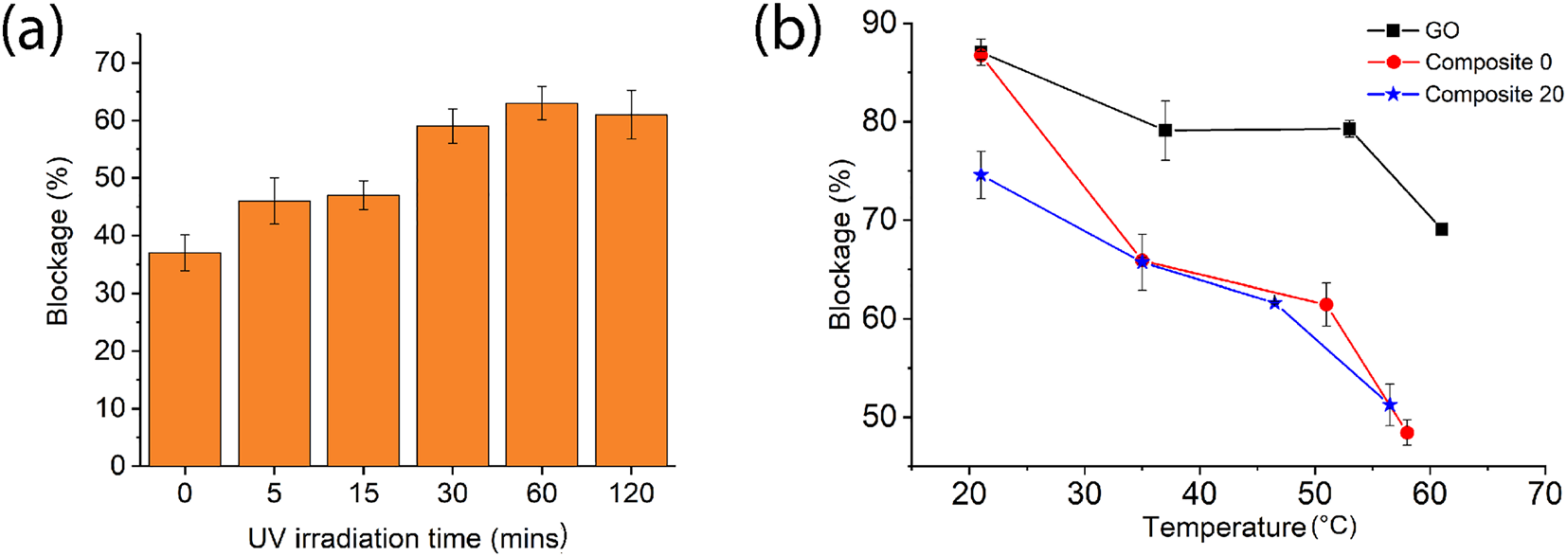
(**a**) Ion blockage percentage of the Composite 0 membranes after exposure to UV light for 0–120 min (n = 2). Ion blockage was measured at room temperature and pH 9. (**b**) Ion blockage percentage of the GO and Composite 0–20 membranes from 22 to 60 °C after 6 h. Data presented as mean ± SD, n = 3.

The ion blockage percentage of the Composite 0 membranes as a function of UV exposure is shown in Fig. 5a. Results show that the blockage percentage practically doubled to ~ 60% after 30 min of UV radiation. The ion blockage performance of the membranes as a function of the filtration temperature is shown in Fig. 5b. The data shows that at pH 4.2 the salt blockage percentage reduces with temperature for all membranes. The reduction is however more pronounced for Composite 0–20 membranes, for which the blockage percentage decreased from 85% at room temperature to less than 50% at 60 °C. The reduction in blockage percentage of the pristine GO membranes decreases by only 15% over this temperature range.

## Discussion

The water filtration capacity of GO membranes has been the subject of substantial research^23^. Previous work from our group has established procedures for producing ZnO/GO composite membranes using zinc acetate as a precursor^19^. In the current work, zinc acetate was partially substituted with zinc acrylate to modulate interactions between the ZnO nanoparticles and the GO flakes. Interestingly, during the synthesis of the Composite 20–80 membranes, it was realized that the order of addition of zinc precursors (acetate or acrylate) determines whether the ZnO nanoparticles bind to the GO flakes or not. In order to form a sufficiently strong bond between the zinc cations and GO flakes, zinc acetate precursors must be added to the GO dispersion first and the mixture must be adequately stirred before the addition of the zinc acrylate, independent of the concentration ratio of the zinc acetate and acrylate. This order of addition could not be reversed. The corresponding peaks of ZnO were absent in the XRD patterns of the Composite 20–80 membranes prepared in reverse order (data not shown). This observation could relate to the different affinity of the acetate vs. the acrylate as ligands for zinc cations due to differences in pKa. When zinc acetate solution is added to the GO dispersion, positively charged zinc cations bind to the edges of the negatively charged GO flakes before they are oxidized into ZnO nanoparticles. A uniform distribution of the ZnO nanoparticles within the Composite 0–20 membranes is shown in the TEM images of Fig. 2a,b and indicates strong repellent forces between their composite flakes.

In a previous report from our group, rhodamine B was successfully filtered out of an aqueous solution using GO/ZnO composite membranes in a dead-end pressure-driven setup^19^. However, these membranes were unable to separate ions from water. Such a result does not meet expectations as the interlayer spacing of the GO/ZnO membranes was down to 7 Å, which should be sufficiently small to block the small hydrated ions such as Na^+^^4^. This discrepancy could be connected to micro-cracks that sometimes appear across the membrane due to the large external pressure applied in a reverse osmosis filtration process^24^. In a diffusion cell setup, the absence of such a force not only reduces the risk of producing physical damage but also allows one to discover the intrinsic blockage capability of a membrane. In fact, high external pressures can alter the interlayer spacing of the GO membrane due to compaction effects^19,25^. The interlayer spacing of a GO membrane represents the size of the narrow channels that physically block the hydrated ions while allowing the passage of water molecules. This micro-crack problem has previously been detected using a helium leak test^6^. Although this method is highly reliable for finding cracks across the GO membrane, it does not guarantee a crack-free membrane once it gets wet. In the current work, the presence of leaks was tested by simply adding a small amount of rhodamine B to the feed solution and checking for the presence of this dye in the permeate. Rhodamine B was not detected in the permeate suggesting that the composite membranes do not crack or become damaged during 24 h when using a non-pressure driven diffusion cell setup.

The ion blockage percentage of the pristine GO membrane is relatively stable within the pH range of 4.2–9, though decreased at lower and higher pH. This might be due to the different protonation states of the functional groups within the nanochannels in this pH range, which affects the effective pore size of the GO membrane^26^. In extremely acidic condition (pH 1), the blockage percentage of the GO membrane reduces to half of its value in neutral pH. Acidity is expected to have two opposite effects on GO-based membranes. On one hand, the ion rejection percentage declines in the acidic solution due to the lower electrostatic repulsion between the GO flakes and the charged solutes (i.e., the Donnan retention effect)^27^. On the other hand, the nanochannels that exist across the GO membrane become narrower under acidic conditions due to the protonation of carboxyl groups on the edges of GO^26^. Hence, the GO membrane is expected to perform better in an acidic medium according to the size-exclusion principle^28^. The results herein imply that electrostatic repulsion between the GO membrane and negatively charged solutes is the main rejection mechanism under acidic conditions. In extremely basic conditions (e.g., pH 12), the interlayer spacing between the GO flakes is expected to increase substantially^29^ and the hydrated ions rapidly pass through the enlarged nanochannels, which manifests itself by low rejection performance. Comparing the blockage performance of the pristine GO and Composite 0 membranes shows that intercalation of the ZnO nanoparticles, produced using zinc acetate, into the lamellar structure of the GO membrane does not significantly affect performance.

Moreover, under most conditions tested, only small and non-systematic differences (if any) were observed between the pristine GO and the Composite 0–60 membranes at the different pH values tested. It should be noted that while dissolution of ZnO nanoparticles was anticipated at pH 1 (but not at pH 12 under the conditions tested) it did not have an obvious effect on blockage, which is consistent with the rest of the dataset showing that the presence of ZnO nanoparticles does not (with the exception below) influence blockage. Indeed, under most conditions, blockage performance was insensitive to the nature of the acrylate vs. acetate ligands on the surface properties of the ZnO nanoparticles. This, however, was not true at pH 12 where the blockage improved from 29% for the Composite 0 membrane to ~ 70% for the Composite 20–60 membranes, irrespective of the content of acrylate. Such a big difference for the membranes containing acrylate groups could reflect the possible reaction of the double bonds within the nanochannels with hydroxide ions (Michael-addition), possibly altering the hydrophilicity and charge state within these channels and hence their sieving properties. However, elucidating such reactions is not trivial and a detailed characterization of the underlying phenomenon related to this large difference as alkaline pH remains is warranted in future work.

Reducing the number of oxygen functional groups on the GO membrane is another way of enhancing its filtration performance^30^. However, the reduced GO membranes are less mechanically robust and usually crack before or during the filtration process^31^. To prevent micro-crack formation, the Composite 0 membranes were kept in a transparent plastic bag right after preparation and during UV treatment. As a result, the partially reduced Composite 0 membranes maintain their integrity for 60 min of UV treatment. The corresponding blockage percentage increased with UV irradiation time, reaching a plateau after ~ 30 min, though cracks were easily observable for over-reduced membranes (120 min; image not shown). Controlled photocatalytic reduction of the Composite 0 membranes shows that its ion blockage performance can be improved to 66% in a non-pressure-driven filtration setup. Unfortunately, the partially reduced membranes cannot withstand the pressure that is required in a reverse osmosis setup. Therefore, additional work is required for the mechanical enhancement of reduced Composite 0 membranes for practical applications.

The filtration properties of the GO-based membranes were reported at various temperatures using a pervaporation setup^32,33^. The total permeation flux increased with operation temperature due to stronger driving forces. In the current work, it is expected that ion permeation increases with temperature based on the theory of Brownian motion and water viscosity. The dynamic viscosity of water at 60 °C is almost half of its value at 20 °C. Therefore, the feed water flows faster at a higher temperature. So it is not surprising that the blocking performance of the membranes reduces with temperature. However, according to Fig. 5b, the rate of blockage is not the same for the pristine GO and Composite 0–20 membranes. The pristine GO membrane performs better over a wide range of temperatures in comparison to the Composite 0–20 membranes. This result could be reconciled by the fact that the pristine GO membrane is substantially more compact and organized than the composite membranes (Fig. 1a–c), which would make it less sensitive to temperature-induced structural changes.

In summary, the positive impact of the acrylate groups on the ion blockage performance of the composite GO membrane in extreme basic conditions is experimentally demonstrated. The TEM images show that utilizing zinc acrylate precursors to synthesize the ZnO nanoparticles had no effect on their crystallization or distribution across the GO membrane. According to the results, the salt blockage percentage of the membranes can be increased to a certain extent, though below that which is considered acceptable for desalination applications. The non-pressure-driven method implies that the filtration imperfections are due to structural imperfections of the GO membranes and not due to applied external pressure. We showed that the shortcoming is more pronounced in warm and acidic feed water.

Furthermore, the results show that the ion blockage capability of photocatalytically reduced Composite membranes can be increased to a certain degree; however, over-reduced GO-based membranes suffer from low mechanical robustness, which must be addressed before their commercialization. Moreover, pristine GO membranes preserved their blockage capability at higher temperatures to a greater extent than composite membranes. This means that while the inclusion of an element may improve one feature of the composite membrane, the performance of another may suffer. Running a comprehensive series of permeation tests under diverse variables (e.g., pH and temperature) for an extended period of time (> 6 h) is therefore warranted to shed light on all features of a novel composite membrane.

## Methods

### Materials

Natural graphite flake, − 10 mesh 99.9% was purchased from Alfa Aesar. Sulfuric acid 95–98%, phosphoric acid 85%, potassium permanganate 99%, hydrogen peroxide solution 30%, zinc acetate dihydrate 98%, copper(II) sulfate 99%, sodium hydroxide 97%, hydrochloric acid 37%, poly(vinyl alcohol) (PVA) 99%, and potassium hydroxide 85% were purchased from Sigma-Aldrich. Polyethersulfone (PES) membrane filters (0.2 μm pore size; 47 mm diameter, 110–150 µm thickness) were purchased from Sterlitech.

### Synthesis of GO and fabrication of the composite membranes

GO flakes were synthesized according to a modified Marcano method, which is more environmentally friendly than the Hummer method and provides a larger amount of hydrophilic GO^34^. 0.5 g of relatively large graphite flakes (~ 2 mm mean diameter) were added to a mixture of 90 mL of sulfuric acid and 10 mL of phosphoric acid under stirring in an ice bath. After 30 min, 3 g of potassium permanganate was added gradually to the mixture under stirring. The mixture was stirred for 2 h at room temperature. To complete the oxidation, the temperature was increased to 55 °C for 24 h. After this period, the mixture was cooled in an ice bath, and 300 mL of nanopure water and 1 mL of hydrogen peroxide were added until a golden mixture was obtained. The GO mixture was washed multiple times with nanopure water via dispersion/centrifugation (7000 rpm) until the pH turned 5. The GO powder was then recovered by lyophilization and stored in a desiccator until used.

To fabricate the GO membrane, 0.012 g of GO powder was dispersed in 30 mL of nanopure water and transferred onto a PVA-treated PES support membrane by vacuum filtration (10 Psi). Treating one side of the PES membrane (the side where the GO sits) with 1 wt% PVA solution improves the physical adhesion of the GO layer to the support membrane, without affecting its permeability^35^. Therefore, one side of the PES membrane was left in contact with the PVA aqueous solution for 1 h, and excess PVA was subsequently removed by rinsing with nanopure water. For the fabrication of the composite membrane, 0.012 g of GO powder was dispersed in 10 mL of methanol. In the second step, 0.22 g of zinc acetate was dissolved in 10 mL of methanol and subsequently added to the GO dispersion under stirring. The mixture was stirred for 30 min and then 0.11 g of KOH, separately dissolved in 10 mL of methanol, was added to the mixture. The mixture was then stirred for 24 h. Methanol was replaced by nanopure water through three consecutive centrifugation/dispersion steps. The final mixture was vacuum-filtered onto a PVA-treated PES membrane as above. To modulate the properties of the composite membrane, zinc acetate was partially replaced with zinc acrylate according to Table 1. The order in which the precursor is introduced to the GO dispersion is very important. In the present case, the zinc acetate solution was first added to the GO dispersion followed by the addition of the zinc acrylate solution. To photo-reduce the Composite 0 membranes, a UV lamp with peak emission at 367 nm and a power of 21 mW/cm^2^ was positioned at a distance of 15 cm above the membrane, which was kept in a sealed transparent acrylic polymer plastic bag to preserve the membrane’s as-prepared humidity. Exposure to UV was maintained for a period of 0–120 min.

**Table 1:** Mass ratio of zinc acrylate and zinc acetate used in the synthesis of the composite membranes.

Sample ID	Zinc acrylate (%)	Zinc acetate (g)	Zinc acrylate (g)
Composite 0	0	0.22	0
Composite 20	20	0.176	0.044
Composite 40	40	0.132	0.088
Composite 60	60	0.088	0.132
Composite 80	80	0.044	0.176

### Characterization

The top surface and cross-section of the membranes were imaged using a Tescan Vega-3 scanning electron microscope (SEM). Elemental analysis was obtained using a Quantax 100 EDS system. The crystallinity of the ZnO nanoparticles within the composite membranes was verified using the Bruker D8 Advance X-ray diffractometer (XRD). The patterns were recorded in the range of 30°–37°, 2θ degrees with a 0.02° step size. The integration of the ZnO nanoparticles into the GO layer was visualized by transmission electron microscopy (HRTEM, JEOL 2100F, 200 kV). The zeta potential of dispersions of pristine GO and Composite 0–60 in water were measured by Zetasizer nano-Z in the pH range 1–12. Measurements were performed three times and the averages are reported. The ion rejection performance of the membranes was measured minimally twice for each membrane using a custom diffusion cell consisting of two 10-mL chambers separated by a 1-cm diameter orifice that holds the membrane. The chambers were filled with 8.5 mL of either a CuSO_4_ solution (3 g/L; feed) or distilled water (permeate). The GO membrane faces the feed solution in this apparatus. To investigate the effect of pH on the blockage performance of the membrane, the pH of the feed chamber was adjusted with concentrated sodium hydroxide or HCl. The pristine GO and Composite 0–60 membranes were tested at room temperature over a 24-h period.

The blockage performance of the UV-exposed Composite 0 membranes was measured as above at room temperature with a feed solution of pH 9. The effect of temperature on the ion rejection performance of the pristine GO and Composite 0–20 membranes was tested between 20 and 60 °C with a feed solution of pH 4.2 over 6 h. The conductivity of both the feed and permeate solutions was measured with an Oakton Con 11 conductivity meter at the beginning and the end of each run. The ion blockage percentage was calculated using the following equation:

$$R = \left( {1 - \frac{{C_{permeate} }}{{C_{average\, feed} }}} \right) \times 100$$

where *C*_*permeate*_ is the conductivity of the permeate chamber after the test and *C*_*average feed*_ is the average conductivity of the feed at the beginning and the end of the test.

## Data Availability

The datasets used and/or analysed during the current study are available from the corresponding author upon reasonable request.

## References

[1] Nair R, Wu H, Jayaram P, Grigorieva I, Geim A (2012). Unimpeded permeation of water through helium-leak–tight graphene-based membranes. Science.

[2] Castelletto S, Boretti A (2021). Advantages, limitations, and future suggestions in studying graphene-based desalination membranes. RSC Adv..

[3] Abraham J (2017). Tunable sieving of ions using graphene oxide membranes. Nat. Nanotechnol..

[4] Mi B (2014). Graphene oxide membranes for ionic and molecular sieving. Science.

[5] Huang H (2013). Ultrafast viscous water flow through nanostrand-channelled graphene oxide membranes. Nat. Commun..

[6] Joshi R (2014). Precise and ultrafast molecular sieving through graphene oxide membranes. Science.

[7] Sun P (2012). Selective ion penetration of graphene oxide membranes. Acs Nano.

[8] Zheng S, Tu Q, Urban JJ, Li S, Mi B (2017). Swelling of graphene oxide membranes in aqueous solution: Characterization of interlayer spacing and insight into water transport mechanisms. ACS Nano.

[9] Yeh C-N, Raidongia K, Shao J, Yang Q-H, Huang J (2015). On the origin of the stability of graphene oxide membranes in water. Nat. Chem..

[10] El Meragawi S, Panda MR, Jovanović P, Majumder M (2023). Current challenges and approaches for energy-efficient ion-selective two-dimensional graphene-based channels: Current approaches for ion selective 2D channels. Curr. Opin. Chem. Eng..

[11] Hu M, Mi B (2013). Enabling graphene oxide nanosheets as water separation membranes. Environ. Sci. Technol..

[12] Zhang M (2020). Molecular bridges stabilize graphene oxide membranes in water. Angewandte Chemie.

[13] Austria HFM (2023). Tailoring the specific crosslinking sites of graphene oxide framework nanosheets for controlled nanofiltration of salts and dyes. J. Clean. Prod..

[14] Lai G (2018). Tailor-made thin film nanocomposite membrane incorporated with graphene oxide using novel interfacial polymerization technique for enhanced water separation. Chem. Eng. J..

[15] Wei, Y. *et al.* Facile and extensible preparation of multi-layered graphene oxide membranes with enhanced long-term desalting performance. *J. Membrane Sci.* 119695 (2021).

[16] Zhang W (2019). One-step sonochemical synthesis of a reduced graphene oxide–ZnO nanocomposite with antibacterial and antibiofouling properties. Environ. Sci. Nano.

[17] Zhong L, Yun K (2015). Graphene oxide-modified ZnO particles: Synthesis, characterization, and antibacterial properties. Int. J. Nanomed..

[18] Wang J (2012). Reduced graphene oxide/ZnO composite: Reusable adsorbent for pollutant management. ACS Appl. Mater. Interfaces.

[19] Hassanpour A (2020). Photocatalytic interlayer spacing adjustment of a graphene oxide/zinc oxide hybrid membrane for efficient water filtration. Desalination.

[20] Liu J (2018). Photo-enhanced antibacterial activity of ZnO/graphene quantum dot nanocomposites. Nanoscale.

[21] Sun P (2014). Selective ion transport through functionalized graphene membranes based on delicate ion-graphene interactions. J. Phys. Chem. C.

[22] Sun P (2015). Highly efficient quasi-static water desalination using monolayer graphene oxide/titania hybrid laminates. NPG Asia Mater..

[23] Wu W, Shi Y, Liu G, Fan X, Yu Y (2020). Recent development of graphene oxide based forward osmosis membrane for water treatment: A critical review. Desalination.

[24] Yang Y (2019). Large-area graphene-nanomesh/carbon-nanotube hybrid membranes for ionic and molecular nanofiltration. Science.

[25] Fernández-Márquez M (2022). Improvement of water filtration performance of graphene oxide membranes on Nylon support by UV-assisted reduction treatment: Control of molecular weight cut-off. Chem. Eng. J..

[26] Zhao G, Zhu H (2019). Self-regulating cross-linked graphene oxide membranes with stable retention properties over a wide pH range. Adv. Mater. Interfaces.

[27] Hu C (2018). Enhancement of the Donnan effect through capacitive ion increase using an electroconductive rGO-CNT nanofiltration membrane. J. Mater. Chem. A.

[28] Huang H (2013). Salt concentration, pH and pressure controlled separation of small molecules through lamellar graphene oxide membranes. Chem. Commun..

[29] Jiang Z (2013). Facilitating the mechanical properties of a high-performance pH-sensitive membrane by cross-linking graphene oxide and polyacrylic acid. Nanotechnology.

[30] Pei J, Zhang X, Huang L, Jiang H, Hu X (2016). Fabrication of reduced graphene oxide membranes for highly efficient water desalination. RSC Adv..

[31] Huang H-H, Joshi RK, De Silva KKH, Badam R, Yoshimura M (2019). Fabrication of reduced graphene oxide membranes for water desalination. J. Membrane Sci..

[32] Halakoo E, Feng X (2020). Layer-by-layer assembly of polyethyleneimine/graphene oxide membranes for desalination of high-salinity water via pervaporation. Sep. Purif. Technol..

[33] Huang K (2014). A graphene oxide membrane with highly selective molecular separation of aqueous organic solution. Angewandte Chemie.

[34] Marcano DC (2010). Improved synthesis of graphene oxide. ACS Nano.

[35] Januário EFD, Vidovix TB, Calsavara MA, Bergamasco R, Vieira AMS (2022). Membrane surface functionalization by the deposition of polyvinyl alcohol and graphene oxide for dyes removal and treatment of a simulated wastewater. Chem. Eng. Process. Process Intensif..

